# Preoperative CT simulation of iliosacral screws for treating unstable posterior pelvic ring injury

**DOI:** 10.1186/s12891-022-05155-6

**Published:** 2022-03-08

**Authors:** Peishuai Zhao, Xiaopan Wang, Xiaotian Chen, Jianzhong Guan, Min Wu

**Affiliations:** Department of Orthopaedics, The First Affifiliated Hospital of Bengbu Medical College, Bengbu, China

**Keywords:** Pelvic injury, Computed tomography, Iliosacral screws, Preoperative planning

## Abstract

**Background:**

The percutaneous iliosacral screw is a common procedure for treating pelvic posterior ring instability. Traditional X-ray fluoroscopy screw placement has the advantages of decreased bleeding and trauma, but it also has some drawbacks, such as increased radiation exposure and screw dislocation. The purpose of this study was to establish a safe, effective, and quick approach for putting iliosacral screws for the treatment of unstable posterior pelvic ring damage utilizing simulated screws based on preoperative computed tomography (CT) planning.

**Methods:**

From February 2019 to June 2020, we retrospectively assessed 41 patients with posterior pelvic ring instability who were treated with percutaneous iliosacral screws in our institution, and randomly separated them into two groups: conventional surgery (*n* = 20) and preoperative planning (*n* = 21). Pelvic radiographs (anteroposterior, inlet, outlet), as well as normal CT scans of the pelvis, were all taken postoperatively to confirm the screw position. After that, the screw insertion time, the radiation exposure time, and the screw misplacement rate (as assessed by postoperative CT) were all examined. Screw position grading was evaluated by Smith grading.

**Results:**

In the conventional surgery group, 26 screws were inserted in 20 patients, with each screw insertion taking 23.15 ± 4.19 min and 1.02 ± 0.17 min to expose to radiation. Eight of the 26 screws were misplaced (30.8%). In the preoperative planning group, 24 screws were inserted in 21 patients, with each screw taking 19.57 ± 4.05 min to implant and 0.67 ± 0.09 min to expose to radiation. One of 24 screws was misplaced (4.2%). Screw insertion time, radiation exposure time, and screw dislocation rate were all significantly reduced when preoperative planning aided iliosacral screw placement (*P* < 0.05).

**Conclusions:**

Preoperative CT simulation of iliosacral screws for placement planning, screw trajectory, and intraoperative screw placement is a safe way for reducing surgical time, radiation exposure, and ensuring accurate screw placement.

## Background

Pelvic fractures caused by high-energy injuries are becoming increasingly common as the social economy increase [1]. The purpose of pelvic fracture treatment is to recover the pelvic ring structure's biochemical stability, allow for early mobility, and reduce the risk of trauma complications [1–6]. Because of the benefits of reduced trauma, less bleeding, rapid recovery, and high biomechanical stability, percutaneous iliosacral screw fixation has increasingly become the standard surgical approach [7].

The usual approach of iliosacral screw placement is to use standard lateral radiographs of the sacrum to establish the insertion point during the procedure and then adjust the direction of the guidewire based on the pelvic inlet and outlet views [4, 5, 8]. This procedure is more reliant on the surgeon's surgical experience and hand feel, and repeated fluoroscopy throughout the surgery may cause the guidewire to lose its position, exposing the surgeon and the patient to additional radiation [6, 9]. Because of the complex three-dimensional anatomical structure of the pelvis, screw dislocation during screw implantation is extremely prone to cause vascular and nerve damage [10]. The precision of screw insertion was considerably impacted by the opacity of intraoperative X-ray fluoroscopy induced by obesity, intestinal gas buildup, and sacral dysmorphism [11]. According to some studies, even though intraoperative X-ray fluoroscopy showed that the screw was completely located in the bone channel, postoperative CT verification revealed that the screw cut out the sacral foramen, and the screw may still be completely dislocated if only intraoperative fluoroscopy is used [5, 12]. Intraoperative 2D navigation, 3D navigation, CT navigation of iliosacral screw insertion have achieved good clinical efficacy and high screw accuracy in recent years [1, 13–15]. However, the costly expense of the equipment prevents it from being widely used in medical care [3].

Preoperative high-quality images are now easier to obtain because updated instruments, and proper preoperative planning can minimize operation time and raise surgeon confidence by evaluating preoperative 3D reconstruction [11]. We used preoperative CT scan data to simulate optimal screw trajectories according to the patient's fracture type to decrease operation time and intraoperative radiation exposure and implant iliosacral screws more accurately. To guide the placement of iliosacral screws during surgery, standard lateral views of the sacrum, pelvic inlet, and outlet views were simulated, and the radiographs were reasonably divided on the S1 vertebral body, and the entry and exit points of the screws were marked on the portions. Through preoperative planning, the aim is to implant iliosacral screws more safely, correctly, and rapidly without the need for specialized equipment or putting a further cost burden on patients.

## Methods

### Patients

This study was approved by the ethics committee of Bengal Medical College (Number: BYYFY-2019KY03). From February 2019 to June 2020, a total of 124 patients with pelvic fractures were treated in our hospital. The following were the inclusion criteria: 1. Age > 14 years; 2. Injury posterior pelvic ring instability (Tile B or C injury); 3. Sacral fracture: Denis zone 1 or zone 2; 4. Pelvic fractures may be minimized by preoperative or intraoperative traction. Exclusion criteria include: 1. Age < 14 years; 2. 0pen pelvic fracture; 3. Having a skin infection in the incision location. 4. Surgical incision and investigation are indicated when vascular and nerve damage is present. 41 patients with pelvic fractures who satisfied the inclusion criteria were divided into two groups at random: the traditional surgery group had 20 patients, whereas the preoperative planning group had 21.

According to the patient's ante-posterior, inlet and outlet X-rays, and a preoperative thin CT scan (0.625 mm thickness), the fracture type was diagnosed. The surgical procedure was determined based on the type of fracture.

All of the patients were supine for the procedure, which was carried out by the same skilled surgeon. The physician determined and utilized standard intraoperative lateral, inlet, and outlet radiographs of the pelvis. Simultaneously, a skilled radiologist operates the same C-arm machine and adjusts it to seek the optimal X-ray image.

According to the standards of screw position published by Smith et al. [16], the screw position was divided into four grades: grade 0, no perforation; grade 1, perforation less than 2 mm; grade 2, perforation of 2–4 mm; grade 3, perforation > 4 mm.

Gender, age, fracture type, mechanism of injury, time of screw placement (from the insertion of the guidewire into the skin to the completion of the screw placement), time of radiation exposure during screw placement, and postoperative screw position were all recorded.

### Procedure

#### Preoperative planning group

**Fig. 1:**
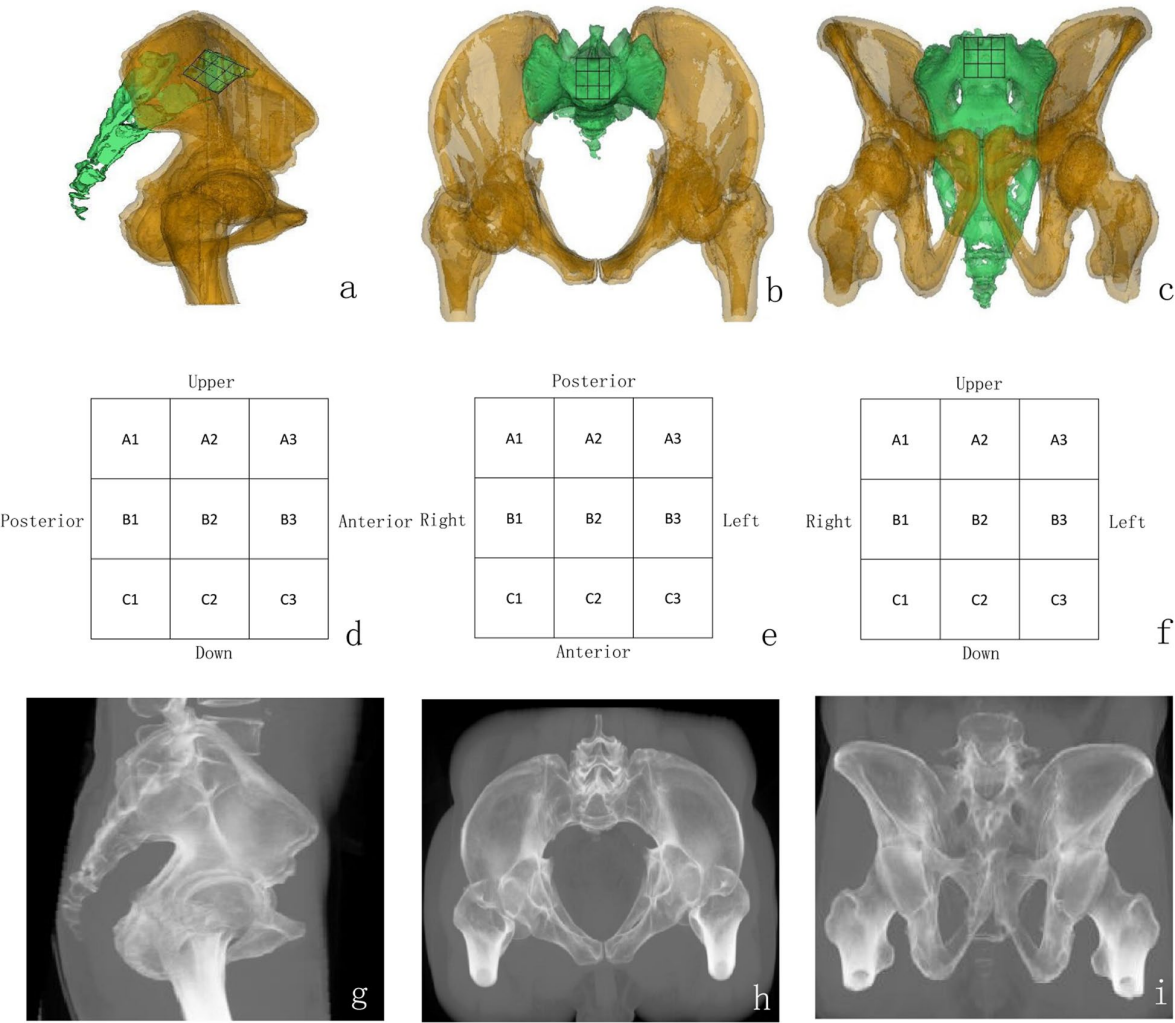
**a** Simulated three-dimensional lateral view reconstruction of the sacrum (Lateral view, L) based on the patient's preoperative CT data; **b** Inlet View (I); **c** Outlet View (O); **d** The sacral 1 vertebral body was planned into 9 regions based on the standard lateral sacral image; **e** The upper endplate of the sacral 1 vertebral body was divided into 9 regions based on the pelvic inlet image; **f** The anterior surface of sacral 1 vertebral body was divided into 9 regions based on the pelvic outlet view, with the medial edge of bilateral sacral 1 anterior foramen as the parallel tangent line of the middle sagittal line and the upper edge of bilateral sacral 1 anterior foramen as the parallel tangent line with the horizontal line; **g** Simulated standard lateral X-ray images of the sacrum; **h** Simulated standard inlet; **I** Simulated standard outlet X-ray image

After admission, the patient underwent a routine plain pelvic CT (Light speed VCT, GE, America) scan, and CT images of 0.625 mm thickness were obtained. CT images were imported into Mimics software (Materialise, Belgium) to obtain the patient's pelvic standard lateral views, and inlet and outlet views (Fig. 1a, b, c). The screw diameter was set to 6.5 mm using the Multiplanar reconstruction (MPR) function in the program to simulate the screw; a screw track was built according to the patient's situation, with the screw at a safe distance from the front, back, above, and below the sacrum. The X-ray simulation capability was utilized to model the screw entry and exit points on the conventional sacral lateral image after the screw was set. At the same time, the screw trace was preserved in conventional sacral lateral, inlet, and outlet images. The nine stages were created by artificially dividing standard sacral lateral images, inlet images, and outlet images. Standard lateral radiographs of the sacrum divided the first sacral vertebra into nine grids of 3 × 3 (classification criteria: Three portions of the sacral 1 upper endplate, three parts of the lower endplate, three parts of the prevertebral cortex, three parts of the posterior vertebral cortex, and the corresponding bisection sites of the upper and lower parts, as well as the anterior and posterior parts, were connected; Lateral radiographs(L), Fig. 1d). The inlet image is separated into nine parts artificially: the vertex of bilateral lateral recess was used to draw a sagittal line forward, and the intersection of the anterior border of sacral 1 vertebral body was indicated. The square area encircled by four points was divided into nine regions. (Inlet radiographs(I), Fig. 1e), and the output image split the medial edge of the bilateral sacral foramina as the parallel tangent line of the middle sagittal line and the top edge of the bilateral sacroforamina as the parallel tangent line of the horizontal line into nine grids. (Outlet radiographs(O) Fig. 1f). On standard lateral views of the sacrum (Fig. 1g), screw entry areas were found, and needle points were marked on the inlet and outlet images in simulated radiography (Fig. 1h, i). According to preoperative planning, screw tracks were simulated on lateral, inlet, and outlet images.

**Fig. 2:**
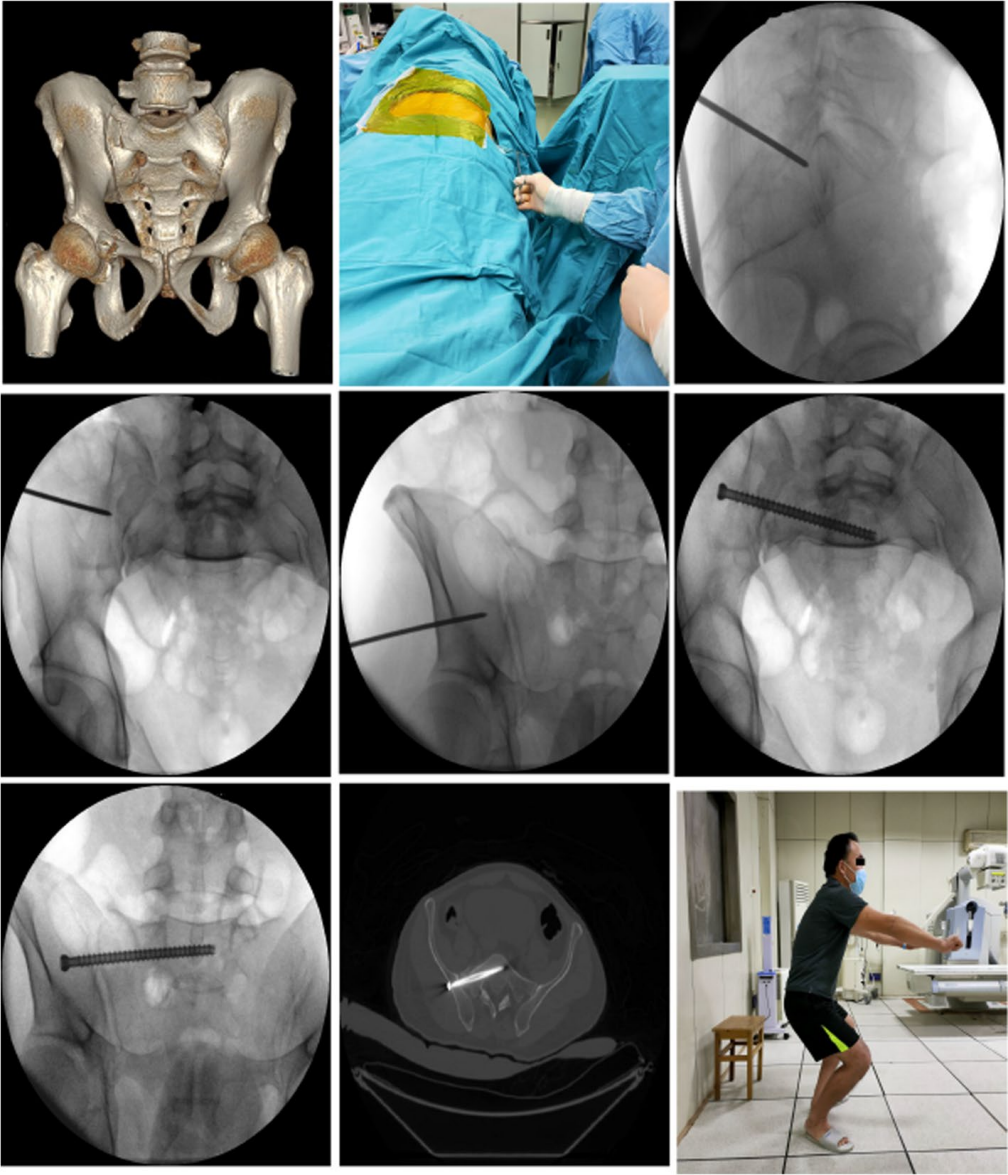
Preoperatively, a 49-year-old male patient involved in a traffic accident was diagnosed with a pelvic fracture (Tile B2). Screw trajectory: sacrum lateral radiographs: insertion point:LC1; Inlet radiograph: screw trajectory:LC1-IC3; Outlet radiograph: screw trajectory:LC1-OB3. **A** Preoperative CT three-dimensional reconstruction of the patient; **B** Intraoperative fluoroscopic positioning screws were used to make needle points in the lateral radiographs of the sacrum; **C** The insertion point of the guide wire was located in the preoperative location area on the lateral radiographs of the sacrum (LC1); **D** The guide needle on the inlet views pointed to the area of the simulated screw exit point before surgery (IC3); **E** The guide needle on the outlet views pointed to the area of the simulated screw exit point before surgery (OB3); **F** Intraoperative inlet radiograph screw trajectory; **G** Intraoperative outlet radiograph screw trajectory; **H** Postoperative CT cross-sectional screw trajectory; **I** The patient can live normally three months following surgery

The iliosacral screw trajectory was generated using the patient's preoperative CT three-dimensional model. The patient's body and the operating table were adjusted so that the patient's body was parallel to the floor at the center of the operating table. After disinfection, the c-arm angle was adjusted to obtain standard lateral and inlet and outlet radiographs of the sacrum. The ground was marked and the rotation angle of the c-arm was recorded. The guiding needle tip was adjusted according to the pre-planned screw insertion location on standard lateral radiographs of the sacrum. The guide needle was gently hammered into the bone using a bone hammer once it had been adjusted to the proper insertion site. At this stage, the inlet and outlet views, as well as the guidewire pointing at the inlet and outlet views, were changed under the preoperative planning. To prevent the guidewire from altering the track, the bone hammer was gradually hammered into the region according to this angle, and fluoroscopy was conducted again after the cortex was penetrated. After the needle was placed along the preoperative trajectory, standard lateral and inlet and outlet radiographs of the sacrum were obtained again. After measuring the required length of the screw with a needle tester, a screw (Synthes Gmbh, Switzerland) of proper length was inserted (Fig. 2).

### Conventional surgery group

**Fig. 3:**
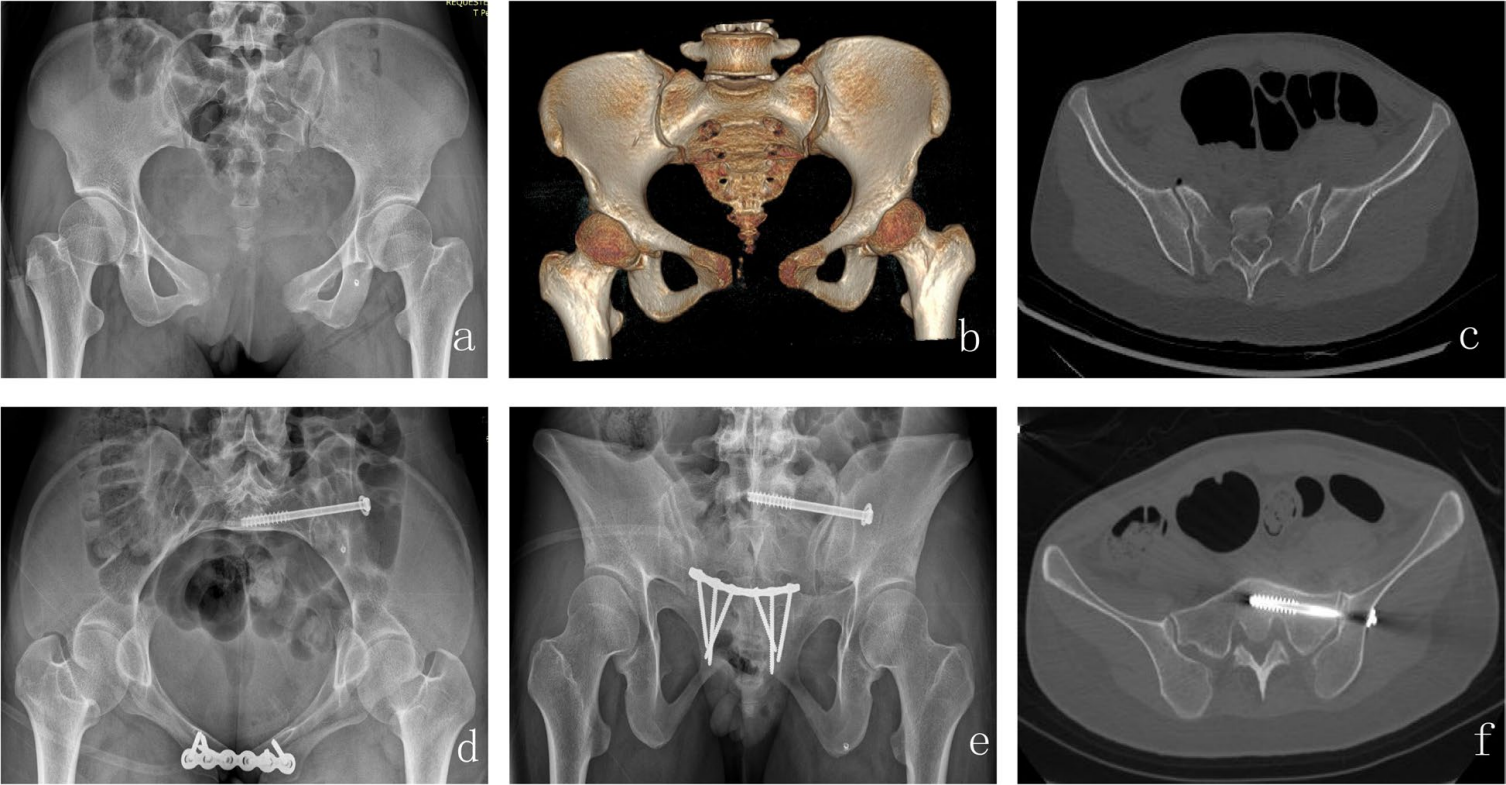
This is a 23-year-old woman who was injured in a traffic accident. The fracture was identified as Tile B1 on the preoperative X-ray. Using conventional X-ray fluoroscopy, the posterior ring was secured using iliosacral screws. **a** Preoperative anterior–posterior view of pelvis; **b** 3D image of pelvic CT; **c** Preoperative CT scan showing left sacroiliac joint separation; **d** Postoperative inlet view; **e** Postoperative outlet view; **f** Postoperative axial CT image

The patient lay supine on a fluoroscopy carbon fiber operating table while the same surgeon used a C-arm machine to execute standard screw placement. The guidewire (diameter: 2.5 mm) was first put in a safe screw placement region after obtaining standard lateral imaging of the sacrum. The guidewire was then carefully hammered into the bone with a bone hammer to prevent the position of the guidewire from being lost. Adjust the guide wire's orientation at the inlet and outlet views, then hammer into it in this direction after it's found in the osseous suitable safe path at the inlet and outlet views. Measure the guide wire's length and insert it in the proper length (Fig. 3).

Anterior–posterior pelvic radiographs, inlet and outlet radiographs, and a postoperative CT scan were taken three days after surgery to determine screw placement and fracture reduction. The quadriceps femoris muscle in both lower limbs was intensively trained on the second day after surgery. One week after a type B fracture, the patient was able to sit up in bed. 2 weeks after surgery: partial weight-bearing; 3 weeks after surgery: full weight-bearing Sit up in bed two weeks after a type C fracture operation; partial weight-bearing four weeks after surgery; full weight-bearing six weeks after surgery. Patients returned to the hospital 1.5, 3, 6, and 12 months following surgery for follow-up.

#### Statistics

Following data collection, t-test and Chi-square tests were performed to compare the traditional surgery group to the preoperative planning group in terms of screw placement time, radiation exposure time, and precision. For categorical data, descriptive statistics are reported as ratios, and for continuous variables, mean plus standard deviation. Fisher's exact test was used to generate p values whenever the anticipated numbers of cell entries were less than 5. SPSS statistics software version 22.0 (SPSS, Chicago, IL) was used for all statistical analyses. Statistical significance was defined as *p*-value < 0.05.

## Results

There were no statistically significant differences between the two groups in demographic or preoperative characteristics (Table 1). There were 11 males and 9 females in the traditional surgery group, with an average age of 43.1 ± 13.0 years. There were 13 cases of traffic accident injuries, three cases of falling from a great height, three cases of crush injuries, and one case of other reasons among them. There were 14 B-type fractures and 6 C-type fractures, according to Tile classification. The preoperative planning group consisted of 9 males and 12 women, with an average age of 41.4 ± 16.1 years. 11 were injured in traffic accidents, five were injured by falling from a great height, four were injured by crushing injuries, and one was injured for other reasons. There were 15 B-type fractures and 6 C-type fractures, according to Tile classification [17].

**Table 1:** Detailed demographic information

Parameter	Conventional group(*n* = 20)	Preoperative planning group (*n* = 21)	*P* value
Gender			0.437
Male,n(%)	11(55.0)	9(42.9)	
Female,n(%)	9(45.0)	12(57.1)	
Mean age(year)	43.1 ± 13.0	41.4 ± 16.1	0.716
Mechanism of injury n(%)			0.866
Traffic Accident	13(65.0)	11(52.3)	
Fall	3(15.0)	5(23.8)	
Crash injury	3(15.0)	4(19.0)	
Else	1(5.0)	1(4.9)	
Tile classification n(%)			0.920
B	14(70.0)	15(71.4)	
C	6(30.0)	6(28.6)	

The results for these two groups are shown in Table 2. In the traditional surgery group, 26 screws were implanted in 21 patients. Each screw took 23.15 ± 4.19 min to insert, and 21 patients in the preoperative planning group received 24 screws. The average operation time for each screw was 19.57 ± 4.05 min, with a statistically significant difference (*P* < 0.05) as compared to the traditional surgery group. Each screw in the traditional surgery group received 1.02 ± 0.17 min of radiation exposures. Each screw in the preoperative planning group received 0.67 ± 0.09 min of radiation exposure, which was statistically significant difference (*P* < 0.001).

**Table 2:** Characteristics of the operation and the results

Groups	Screws(*n*)	Per screw of operative time(min)	Per screw of radiation exposure(min)
Conventional group	26	23.15 ± 4.19	1.02 ± 0.17
Preoperative planning group	24	19.57 ± 4.05	0.67 ± 0.09
*P*		0.009	< 0.001

**Fig. 4:**
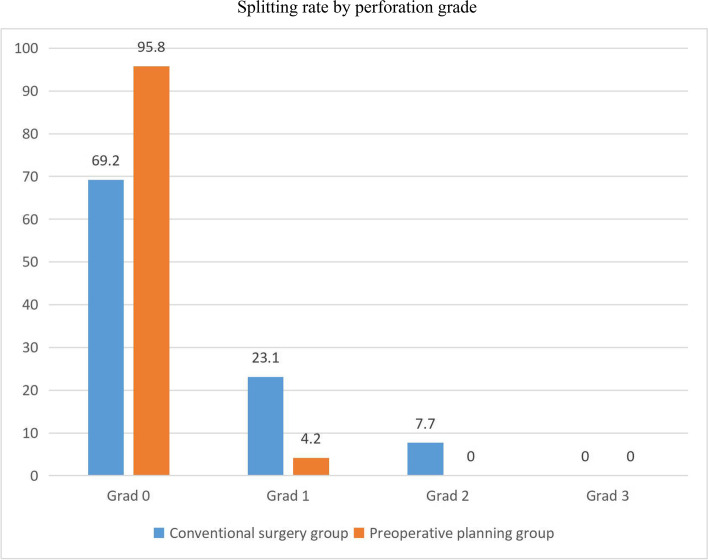
Perforation of screws at the conventional surgery group versus the preoperative planning group (*p* < 0.05)

Eighteen screws (69.2%) were totally inserted in the bone channel in the conventional surgery group, six screws (23.1%) were close to the sacral foramen cortex but did not extend into the sacral foramen, and two screw (7.7%) penetrated the sacral foramen. 23 screws (95.8%) were totally inserted in the bone channel in the preoperative planning group, one screw (4.2%) was close to the cortex of the sacral foramen, and no screws entered the sacral foramen (Fig. 4). There were no signs or symptoms of nerve injury in any of the patients, and no surgical repair was performed.

## Discussion

The majority of orthopedic surgeons utilize percutaneous iliosacral screw fixation as a standard surgical procedure [6, 7, 13, 18]. Letournel et al. [19] used a new mixed open reduction technique to successfully install iliosacral screws in patients with unstable pelvic fractures. Many academics have modified this technique [2, 10]. Matta et al. [20] suggested judging pelvic ring injuries on the pelvic inlet and outlet images and using these images to alter the position of surgical screws based on prior studies. Routt et al. [21] proposed a standard lateral sacrum radiograph, indicating that the safety zone improved the accuracy of iliosacral screw insertion significantly. The iliosacral screw fixation technique is still challenging due to the non-orthogonal planes at the inlets and outlets of the pelvis [22, 23]. During the procedure, changing the orientation of the guiding wire in different views leads the operator and the patient to suffer more exposure to radiation [9]. The report pointed out that the incidence of screw dislocation with traditional fluoroscopy is 2%-15% [9]. This number may be higher in patients  with upper sacral segment deformity [13, 18, 24].

Ebraheim et al. [25] proposed the use of intraoperative CT-guided placement of iliosacral screws based on cadaver studies. The sacral nerve foramen and sacral canal can be observed under the guidance of intraoperative CT fluoroscopy, and screws can be placed, so that the risk of screw penetration greatly reduced. However, use of intraoperative CT fluoroscopy under the nail will make the operator, the operating team, and the patient to bear a larger radiation dose. Gianluca Ciolli et al. [24] used the O-arm navigation system to use low radiation doses to virtual screws during surgery, and could calculate the length of the screw, verify the screw trajectory on the monitor, and then insert the appropriate screw. The average insertion time of each screw was 41.0 ± 12.5 min. The reason for the long time may be related to the preparation of the equipment. This method can achieve a higher accuracy of screw placement, but it is time-consuming and requires high equipment and technology.

Studies have demonstrated that intraoperative CT fluoroscopy navigation and computer 2D/3D navigation can guide screw placement with greater accuracy, but it has not been widely adopted due to the high technical requirements and high overall costs [13, 26–29]. Furthermore, intraoperative CT and 2D and 3D navigation are frequently used to plan screw trajectory during the operation [13, 30]. The traditional nail placement method continues to be widely used in clinical practice, despite the availability of several intraoperative navigation systems [31].

Clinicians face a significant challenge in treating unstable pelvic fractures. First, high-energy injury patients frequently have many injuries throughout the body, and poor blood perfusion caused by large blood loss frequently exacerbates tissue and organ harm [1]. Second, the procedure is difficult and time-consuming, and patients' intraoperative bleeding is a big worry [32]. Finally, a prolonged recovery time has a negative impact on the mental, psychological, and physical well-being of patients [4]. As a result, trauma orthopedic surgeons have made it their mission to simplify surgeries in order to save time, trauma, and surgical difficulties, as well as to ensure a speedy postoperative recovery [3, 33]. There is currently no literature that explains the screw trajectory of iliosacral screws, and the screws are only approximately designed to be totally situated in the bone. From the clinical study, it was discovered that patient variability contributes to insertion point uncertainty, and that varied insertion locations result in a change in the angle and direction of the insertion [5, 18, 34]. This makes surgical screw placement extremely challenging [9, 18]. Preoperative CT and trajectory planning screw into the needle point, as well as software to simulate the sacrum standard side, pelvic inlet, and outlet X-ray radiography, were used to guide intraoperative more accurate placement of iliosacral screws compared to traditional intraoperative fluoroscopy pelvic entrance, and it was decided to screw into the needle track, with less intraoperative fluoroscopy and safer screw placements. The study provides the advantages of simplicity, ease, inexpensive, and equivalent accuracy while requiring no costly surgical equipment. It can minimize the operator's learning curve, lower the danger of screw penetration, and lower the risk of neurovascular injury. This technology still has its shortcomings:1. It requires the same high fluoroscopic clarity as conventional surgery. 2. Screw trajectories have not been planned for every possible screw path.3. Experience as an operating surgeon is essential. 4. Standard sacral lateral views, inlet views, and outlet views are difficult to obtain according to preoperative planning. This technique is challenging for those who perform preoperative planning, and still needs to be improved to be more effective and accurate.

## Conclusion

Preoperative planning assists and guides intraoperative placement of iliosacral screws for the treatment of unstable posterior pelvic ring injuries, simplifying the surgical procedure and permitting surgeons to insert iliosacral screws more safely. It can significantly reduce radiation exposure, shorten operation time, and improve precision when compared to standard surgical approaches. Preoperative planning opens up a novel option for percutaneous iliosacral screw fixation that warrants further investigation.

## Data Availability

The datasets used and/or analysed during the current study are available from the corresponding author on reasonable request

## Abbreviations

CT: Computed tomography
2D: Two-dimensional
3D: Three-dimensional
MPR: Multiplanar reconstruction
